# Pharmacological analysis of Empagliflozin: Acting through the CaMKII pathway in type 2 diabetes and acute cardiovascular events

**DOI:** 10.1371/journal.pone.0270152

**Published:** 2022-06-29

**Authors:** Guangyao Shao

**Affiliations:** Qingdao University, Ri Zhao, Shan Dong Province, China; Universita degli Studi di Torino, ITALY

## Abstract

**Background:**

Type 2 diabetes mellitus is a high-risk factor for acute cardiovascular events. Some reports show that Empagliflozin has a protective effect on cardiovascular events and diabetes mellitus, and Empagliflozin can act on the CaMKII pathway. However, the specific gene of action is not precise. Therefore, this study investigated the target genes of Empagliflozin by integrated gene analysis and molecular docking method to provide a theoretical basis for further elucidating the mechanism of action of Empagliflozin.

**Method:**

In this study, we obtained 12 datasets from GEO, divided into experimental and validation groups, with a total of 376 samples. We then integrated CaMKII pathway-related genes from OMIM, NCBI, and genecards databases. We then intersected them with the differential genes we obtained to obtain 5 common genes and performed functional enrichment analysis. We then performed group comparisons in the validation set, and we obtained 2 clinically significant genes. Then we performed group comparison in the validation set, and we obtained 2 clinically significant genes, followed by molecular docking analysis with pymol, autodock software. We obtained molecular docking models for the 2 genes.

**Conclusion:**

In this study, we obtained *CaMK2G* and *PPP1CA*, genes associated with the CaMKII pathway and type 2 diabetes and acute cardiovascular events, by integrative gene analysis and validated their expression in the relevant dataset. We also derived that Empagliflozin acts on amino acid *TRP-125* of *CaMK2G* gene and *GLN-249 ASP-210 ASP-208* of *PPP1CA* through CaMKII pathway, thus acting on type 2 diabetes and acute cardiovascular events by molecular docking technique.

## Background

Patients with type 2 diabetes are at increased risk of developing cardiovascular disease [1]. Empagliflozin may reduce mortality from cardiovascular events by affecting hemodynamic effects [2]. It has been shown that empagliflozin contributes to cardiovascular disease by acting on the CaMKII pathway, decreasing its activity and reducing CaMKII pathway-dependent calcium efflux [3]. However, the genes in which Empagliflozin acts on the CAMKII pathway have not been reported. The search for markers of Empagliflozin at the transcriptome level is essential to understand the mechanism of action of Empagliflozin. Therefore, this study was conducted to investigate and demonstrate the target genes of Empagliflozin by integrating bioinformatics techniques and molecular docking techniques.

## Method

### Data acquisition and processing

In this study, 12 datasets with a total of 376 samples(The specific information such as the origin of the tissue is shown in Table 1) were obtained from the Gene Expression Omnibus (GEO database, https://www.ncbi.nlm.nih.gov/geo/) [4], and differential gene analysis was performed using R language to draw heat maps and volcano maps. In this study, the data from the GEO database were screened. The screening criteria were as follows: 1. search with the keywords "type 2 diabetes" and "acute heart disease." 2. restrict the entry type to series 3. restrict the study type to expression analysis of arrays. 4. the data retrieved had controlled. 5. the data retrieved had genetic samples. 6. the organisms were restricted to Homo sapiens.

**Table 1:** Information of the dataset used for the study.

GEO_ID	tissue	platform	sample		molecule	country
			control	Disease		
GSE48060	blood	GPL570	21	31(MI)	total RNA	USA
GSE19339	blood vessel	GPL570	4	4(MI)	total RNA	Switzerland
GSE97320	blood	GPL570	3	3(MI)	total RNA	China
GSE12643	skeletal muscle	GPL8300	10	10(T2D)	total RNA	Denmark
GSE13760	arterial tissue	GPL571	11	10(T2D)	total RNA	Denmark
GSE20966	pancreatic	GPL1352	10	10(T2D)	total RNA	USA
GSE25724	islets	GPL96	7	6(T2D)	total RNA	Italy
GSE29231	biopsy samples of visceral adipose	GPL6947	12	12(T2D)	total RNA	India
GSE71416	omental adipose	GPL570	6	14(T2D)	total RNA	USA
GSE78721	adipocytes and infiltration macrophages	GPL15207	62	68(T2D)	total RNA	India
GSE60993	blood	GPL6884	7	10(NSTEMI)	total RNA	South Korea
				7(STEMI)		
				9(UA)		
GSE61144	blood	GPL6106	10	7(STEMI)	total RNA	South Korea
				7(recovered STEMI)		

MI: myocardial infarction.T2D: type 2 diabetes. STEMI:ST-elevation myocardial infarction, NSTEMI:Non-ST-elevation myocardial infarction and UA: unstable angina. recovered STEMI: Blood samples from STEMI patients 7 days after initial percutaneous coronary intervention

Exclusion criteria were as follows: 1. The data retrieved had drug treatment or other confounding factors in the experimental group. 2. The data retrieved did not have genetic samples. 3. The data obtained from the survey did not have grouping information. 4. The data obtained by probing did not have grouping information. 5. The source of the samples was not Homo sapiens.

We screened out the experimental group for differentially expressed gene analysis and the validation group for critical genes. The specific information is as follows (Table 1).

### Data analysis and identification of DEGs

Quality control was performed on the datasets obtained from GEO. Subsequently, the R package "limma" was used to analyze variance between samples. P-values of genes and adj. p-values after hypothesis testing were obtained after correction. The meaning of the corrected P-value is the P-value obtained after hypothesis test calibration [5, 6]. Log2 (fold change) >1.5 or -1.5 and a p-value of 0.05 were used as screening criteria.

To avoid the effect of differences in gene expression in different tissues on the analysis results, we used the original grouping method in the dataset. We performed the analysis of differential gene expression within the original dataset. For example, in the dataset GSE48060, the raw data were obtained from blood and grouped into MI and control groups. We analyzed the raw matrix data into the R language for differential gene expression comparison.

### Heatmap and volcano plot

Heatmaps and volcano maps of differential genes were obtained using the heatmap package in R software.

### Enrichment analysis

Metascape is an online functional enrichment analysis website. It mainly includes interactive enrichment and more [7]. WebGestalt is a tool that is very commonly used for feature enrichment analysis and contains user-uploaded data. It provides researchers with a more convenient way to enrich features [8]. We used the Metascape website and the webgestalt website to perform functional enrichment analysis of the more than 5037 genes we obtained and visualize them to observe the main biological functions of these differential genes and the primary pathways. The search was performed by:1. It entered differential genes as keywords into the search box.2 and limited their species to homo sapiens.3. the Method of Interest in the webgestalt website was limited to over-representation analysis.

### Identification of core genes

In order to obtain the maximum number of differential genes and avoid drug-induced gene count reduction as a confounding factor, our original dataset was not selected for patients using Empagliflozin or not using Empagliflozin. Instead, we selected samples of patients with the disease and normal controls.

The CaMKII Pathway was selected as a screening condition because it has been demonstrated that the CaMKII Pathway may be a pathway for the pharmacological effects of Empagliflozin in type 2 diabetes and acute myocardial infarction. To test the possibility of this hypothesis, we conducted the present study. There are no relevant experiments to illustrate the pharmacological pathway of Empagliflozin in type 2 diabetes mellitus and acute myocardial infarction. Therefore, this study sought to demonstrate the possibility of CaMKII Pathway as a pharmacological pathway of Empagliflozin in type 2 diabetes and acute myocardial infarction.

OMIM (http://omim.org) is an online database of genetic information used by clinicians, molecular biologists, and genomic scientists [9]. GeneCards (www.genecards.org) is an integrated database of gene information. It brings together genetic data from several considerable public resources, such as HGNC [10], NCBI [11], ENSEMBL [12], and UniProtKB [13], as well as many other smaller resources [14]. GeneCards include genomic, proteomic, transcriptomic, disease, and functional data for known human genes [15].

The U.S. National Center for Biotechnology Information (NCBI) is an online database of genetic information. It includes the retrieval and storage of genomic data. It provides free downloadable data to a wide range of researchers [16]. We analyzed the common genes obtained by differential analysis with the CaMKII pathway-related genes obtained by OMIM database NCBI database genecards database. We selected the genes they contained in common, i.e., the intersection was taken.

### Functional enrichment analysis of essential genes

To observe the main biological functions and significant pathways of the five essential genes, we entered these five gene names as keywords into the search box of the Metascape website and webgestalt website. The search was performed by:1. We entered differential genes as keywords into the search box.2, limiting their species to homo sapiens.3. the Method of Interest in the webgestalt website was limited to over-representation analysis. In addition, we obtained upstream miRNAs for five essential genes by using the mRNA-miRNA module of the online miRNA database StarBase (version 3.0; http://starbase.sysu.edu.cn/index.php) [17].

### Verification of the hub genes

We used the dataset GSE60993 GSE61144 as the validation group, extracted the expression of five essential genes, and compared them in groups according to the clinical stage of their disease, using t-tests with p-values as a measure of test effectiveness.

### KEGG pathway analysis

We performed a KEGG pathway analysis to observe the pathways enriched by the five essential genes. KEGG (https://www.kegg.jp/) is a comprehensive database, of which the PATHWAY database is widely used for gene pathway studies [18]. KEGG mapper is a visualization tool for the KEGG database, which allows the visualization of gene enrichment pathways [19]. We entered the names of five critical genes as keywords into the search box of KEGG Mapper and limited the search mode to hsa. We obtained visualization maps of the pathways enriched by five essential genes. Among them, the Diabetic cardiomyopathy pathway and the cAMP signalling pathway should be of most interest to us.

### Molecular docking

Autodock is currently maintained by Scripps Research. autodock is a tool that can perform molecular docking. The study showed that the RMSD (root mean square deviation, a metric that evaluates the sampling power of each program) and prediction accuracy of autodock software are relatively high [20, 21]. We used the autodock tool to perform molecular docking studies to determine the effective molecules of Empagliflozin that could bind to the proteins transcribed from our screened target genes and thus exert their biological effects.

For this purpose, we entered Empagliflozin as a keyword in the PubChem website (http://pubchem.ncbi.nlm.nih.gov, a public database of active ingredients of small molecules and RNAi reagents is available) to retrieve its active molecule as a ligand [22]. We have downloaded the 2D structure of Empagliflozin. The conversion was performed in Chem3D software. The energy-minimized structure of Empagliflozin was calculated and saved using the MM2 method by clicking on the calculation module in the software. The minimum RMS gradient was set to 0.01.We then searched the UniProt database (http://sparql.uniprot.org/, a database for storing protein information) with "CaMK2G" and "PPP1CA" as keywords to obtain the protein files of 1.7A and 2.25A as receptors, respectively [23]. We downloaded the 3D data files of CaMK2G and PPP1CA protein structures.

PyMOL is open-source software for displaying the 3D structure of molecules written by DeLano Scientific LLC. We used pymol software to remove water molecules and ligands from the obtained receptor files. We then used autodock software to add hydrogen atoms and create active pockets and molecular docking. We clicked on the Grid to adjust the range of active pockets of the protein 3D structure.The specific parameters were as follows: the number of points in X dimension was 50, the number of points in Y dimension was 60, the number of points in Z dimension was 52. the spacing was 1, the x center was -20.012, the Y center was -32.916, the Z center was -5.221. the energy range was 5, and the maximum number of models was 20. We then performed a semi-flexible docking approach for molecular docking.

### Statistics analysis

We used the student’s t-test to see any differences between the two groups. We used the rgpubr R package and the ggplot2 R package. rgpubr package was used for calculations, and ggplot2 was used for visualization.

## Results

### Differential gene analysis

**Fig 1:**
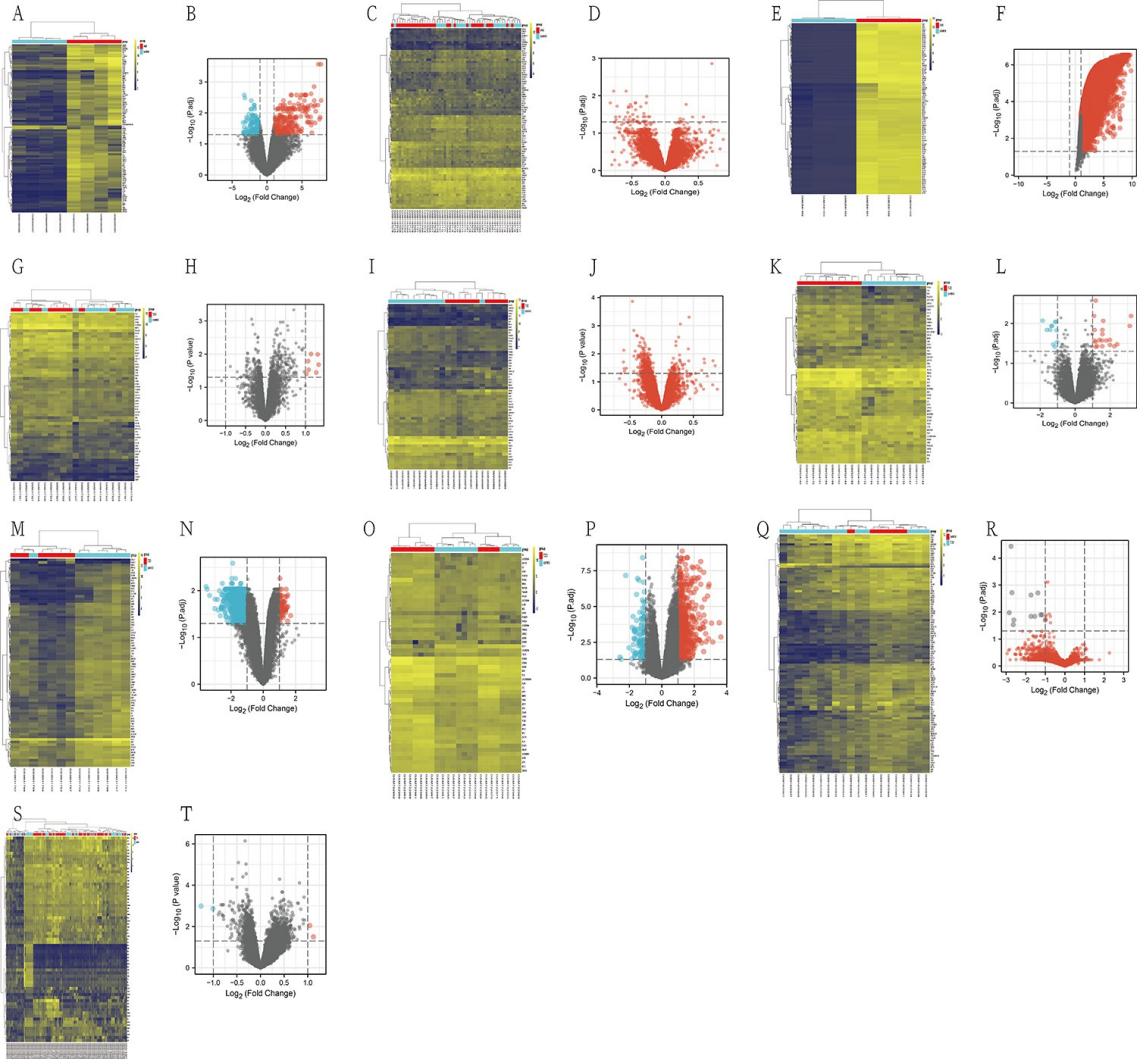
A.the heat map of GSE19339. B.the volcano map of GSE19339. C.the heat map of GSE48060. D.the volcano map of GSE48060. E.the heat map of GSE97320.F.the volcano map of GSE97320. G. the heat map of GSE12643. H.the volcano map of GSE12643. I.the heat map of GSE13760. J.the volcano map of GSE13760. K.the heat map of GSE20966.L.volcano map of GSE20966. M.the heat map of GSE25724. N.volcano map of GSE25724. O.the heat map of GSE29231. P. volcano map of GSE29231. Q.the heat map of GSE71416. R.volcano map of GSE71416. S.the heat map of GSE78721. T. volcano map of GSE78721.

**Fig 2:**
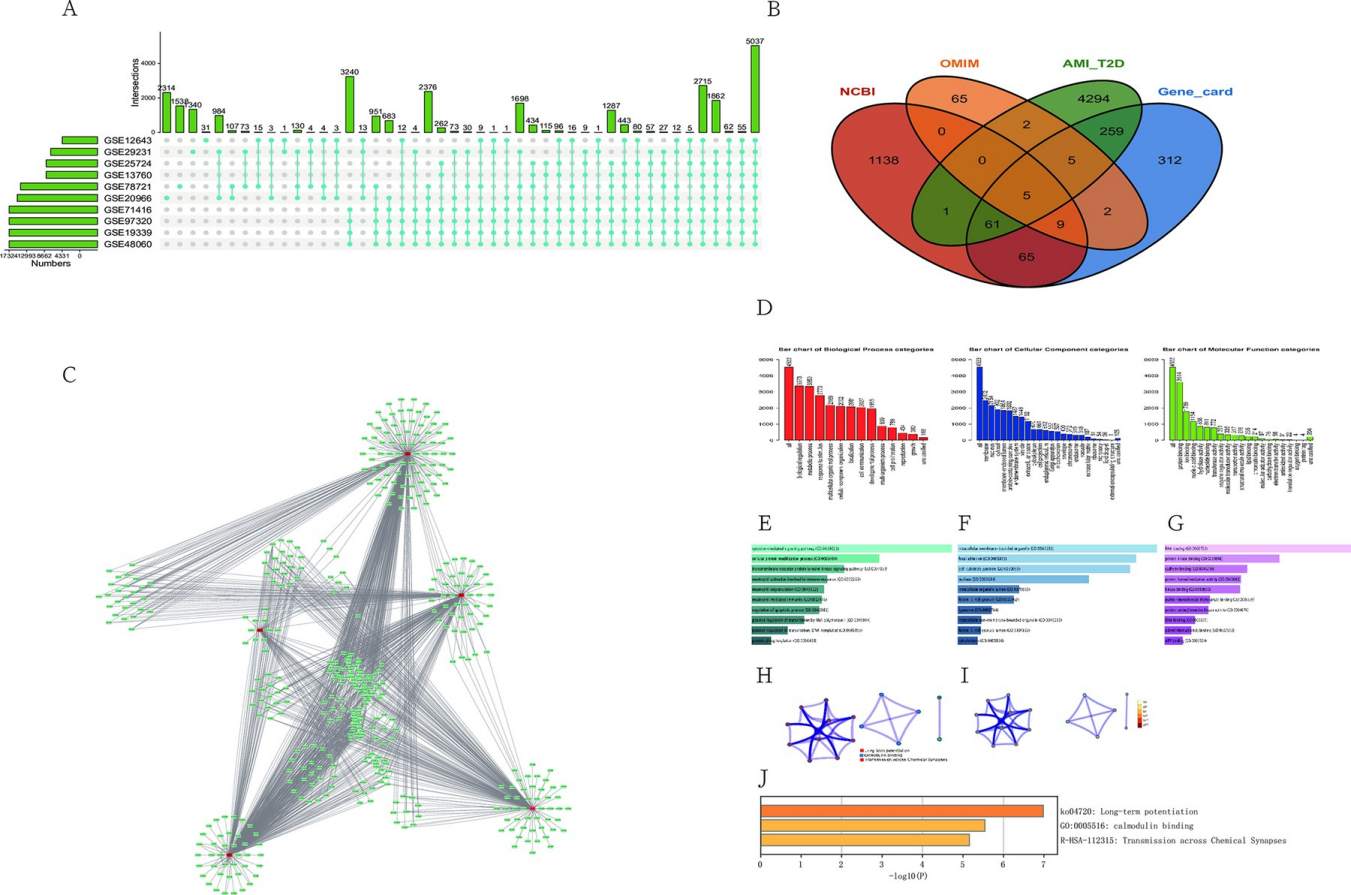
A. UPset plots of the 10 experimental datasets, the blue dots represent the genes in the datasets, the vertical coordinates represent the names of the datasets, and the horizontal coordinates represent the number of genes. Among them, 5037 genes were expressed in all datasets. B. Venn diagram, whose China includes CaMKII-related genes from three NCBI OMIM gene card databases and 4294 differential genes obtained from our analysis using the GEO database. Five genes were common to the four datasets. C. PPI network diagram. Potential upstream miRNAs of 5 critical genes obtained by analysis of the mRNA-miRNA module of the starbase database. D. Molecular function, major cellular components, and biological processes of 5,037 differential genes analyzed by webgestalt database. The main enriched cellular components are the membrane. The primary enriched molecular function is protein binding. The main enriched biological process is biological regulation. E.Bar graphs were obtained from Metascape database analysis. The immediate enriched biological process is cytokine-mediated signalling. F.Bar graphs obtained from Metascape database analysis. The main enriched cellular component is intracellular membrane-bounded organelle. G.Bar charts obtained from Metascape database analysis. The main enriched molecular function is RNA binding. H. Enrichment network map of five critical genes obtained from Metascape database analysis. Notably, the five essential genes were enriched in calmodulin-binding and transmiss on across chemical synapses. I. Enrichment network map of five critical genes obtained from Metascape database analysis. The darker yellow colour represents the higher correlation of the interrelationship of reactions. J. Bar graphs of the five essential genes enrichment obtained from Metascape database analysis.

Quality control was performed on the dataset obtained from GEO. All downloaded datasets were normalized by the R affy package using a robust multi-array average normalization method. Subsequently, differences between samples were analyzed using the R package "limma" and visualized using a heat map package, and we obtained 10 heat maps and volcano maps. Next, we plotted the disturbance maps using the R language. We obtained 5037 shared genes, which means that these 5037 genes intersect the differential genes obtained from the differential analysis of all data sets (Figs 1 and 2A).

### Enrichment analysis

Metascape is an online functional enrichment analysis website. It mainly includes interactive functional enrichment and more [7]. WebGestalt is a tool that is very commonly used for feature enrichment analysis and contains 12 and 155 175 feature categories and user-uploaded data. It provides researchers with a more convenient way to enrich features [8]. We used the Metascape website and the webgestalt website to perform functional enrichment analysis of the more than 5037 genes we obtained and visualize them to observe the main biological functions of these differential genes and the primary pathways. The main cellular component enriched by the 5037 differential genes analyzed through the webgestalt database is the membrane. The primary enriched molecular function is protein binding. The main enriched biological process is bioregulation. The 5037 differential genes analyzed from the Metascape database were mainly enriched for the biological process of cytokine-mediated signalling, and the main enriched cellular component was the intracellular membrane bundle organelle. The main enriched molecular function is RNA binding (Fig 2D–2G).

### Identification of core genes

We analyzed the common genes obtained by differential analysis with the CaMKII pathway-related genes obtained by OMIM database NCBI database genecards database. We selected the genes they contained in common, i.e, the intersection was taken.As shown in the Venn diagram, we obtained 5 shared genes.By taking the intersection of CaMKII-related genes from the three NCBI OMIM gene card databases and the 4294 differential genes we obtained using the GEO database analysis, we obtained five genes (Fig 2B).

### Functional enrichment analysis of essential genes

To observe the main biological functions and significant pathways of the five essential genes,we entered these five gene names as keywords into the search box of the Metascape website and webgestalt website.Five important genes obtained from Metascape database analysis are enriched in calmodulin-binding and trans-chemical synapses. (Fig 2H–2J) In addition, we used the Starbase database to analyze the upstream miRNAs of five essential genes (Fig 2C).

### Verification of the hub genes

**Fig 3:**
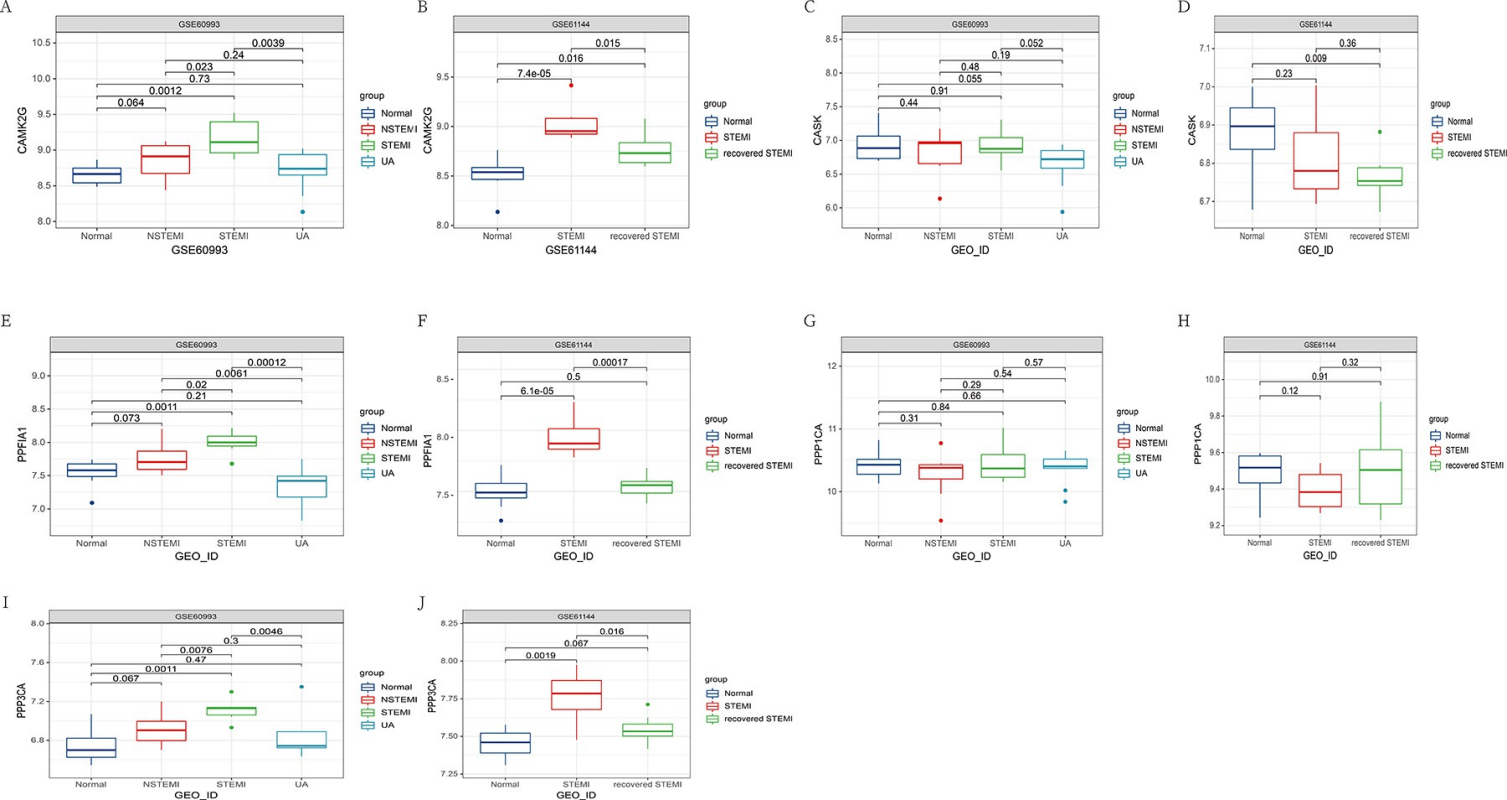
A. Box plot of the expression of CaMK2G in GSE60993. B. Box plot of CaMK2G in GSE61144. C.Box plot of the expression of CASK in GSE60993. D.Box plot of the expression of CASK in GSE61144. E. Box plot of the expression of PPFIA1 in GSE60993. F. Box plot of the expression of PPFIA1 in GSE61144. G. Box plot of the expression of PPP1CA in GSE60993. H. Box plot of the expression of PPP1CA in GSE61144. I. Box plot of the expression of PPP3CA in GSE60993. J. Box plot of the expression of PPP3CA in GSE61144.

We used the dataset GSE60993 GSE61144 as a validation group, extracted their expression about 5 essential genes, and compared them in groups according to the clinical stage of their disease, using a t-test and using the p-value as a measure of the effect of the test, we obtained Fig 3. As shown in the Fig 3, the p-values of *CaMK2G PPP1CA* and *PPFIA1* were less than 0.05, and their expression levels were consistent with the clinical features, so we considered *CaMK2G PPP1CA* and *PPFIA1* as the most significant genes among the five essential genes. Notably, in the box plot of GSE60993, the expression of CaMK2G was higher in acute ST-segment myocardial infarction than in acute non-ST-segment myocardial infarction and higher than in normal myocardium. In the box plot of GSE61144, *CaMK2G* expression was more significant in acute myocardial infarction than in normal myocardium. After recovery, the expression was lower in acute myocardial infarction but still higher than in normal myocardium (Fig 3).

### KEGG pathway analysis

**Fig 4:**
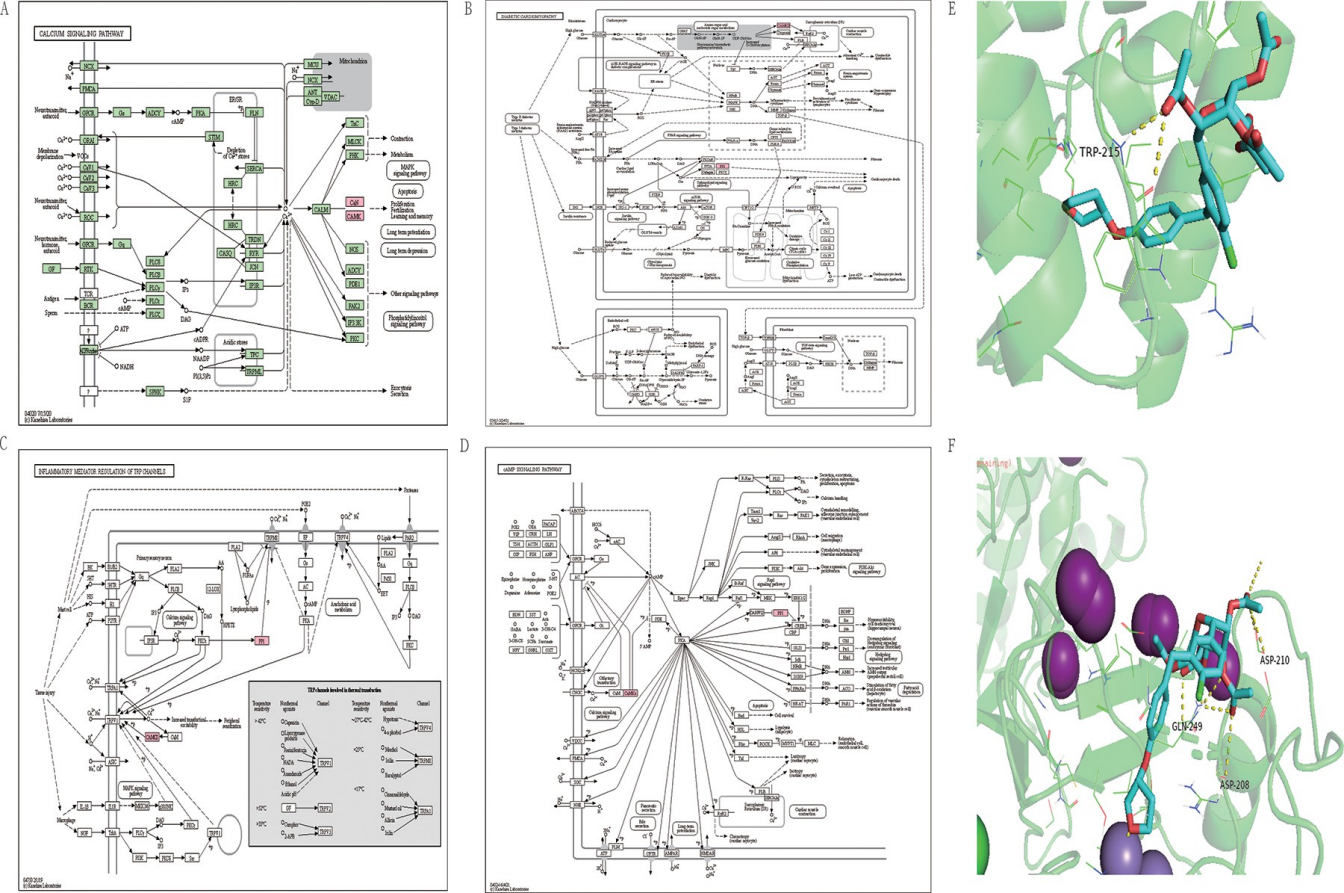
A. KEGG pathway diagram of hsa04020, Calcium signalling pathway. B. KEGG pathway diagram of hsa04024.cAMP signalling pathway. C. KEGG pathway diagram of hsa04750. Inflammatory mediator regulation of TRP channels. D. KEGG pathway diagram of hsa05415.Diabetic cardiomyopathy. E. Molecular docking diagram of CaMK2G and Empagliflozin. Empagliflozin binds to the TRP-215 residue of CaMK2G. F. Molecular docking diagram of PPP1CA and Empagliflozin. Empagliflozin binds to residues GLN-249 ASP-210 ASP-208 of PPP1CA. The purple spheres in the figure are iodide ions, and the green spheres are chloride ions.

We performed a KEGG pathway analysis to observe the pathways enriched by the five essential genes. KEGG (https://www.kegg.jp/) is a comprehensive database, of which the PATHWAY database is widely used for gene pathway studies. kegg mapper is a visualization tool for the KEGG database, which allows visualization of gene enrichment pathways. We entered the names of five critical genes as keywords into the search box of KEGG Mapper and limited the search mode to hsa. Among them, the Diabetic cardiomyopathy pathway and the cAMP signalling pathway should be of most interest to us. Notably, in the CaMP pathway, CNGC forms CaMK and participates in the CaMP pathway, which transfers calcium ions to cells. In the pathway of regulation of TRP channels by inflammatory mediators.CaMKII is involved in regulating TRP channels by inflammatory mediators by interacting with TRPV1 protein.PPP1 is involved in regulating TRP channels by inflammatory mediators through interaction with TRPM8 protein. In the Diabetic cardiomyopathy pathway, CaMKII is involved in myocardial contraction by interacting with RyR2 protein and causing contractile dysfunction. PPP1 is involved in Cardiomyocyte death (Fig 4).

### Molecular docking

We imported the receptor protein from UniProt into pymol software to remove water and ligand molecules. We then imported the new protein receptor file and ligand molecule file into autodock software to perform hydrogenation and molecular docking analysis. The binding energies of the ligands to the *CaMK2G* and *PPP1CA* proteins were -7.6 and -8.6, which means that they require very little power to bind to the ligand molecules, i.e. they bind efficiently to the ligand molecules. In addition, I also show the amino acid residues that bind the ligand molecule to the receptor molecule. Among them, the effective molecule of Empagliflozin binds by hydrogen bonding to residue *TRP-215* of *CaMK2G* protein and by hydrogen bonding to residue *GLN-249 ASP-210 ASP-208* of *PPP1CA* protein (Fig 4E and 4F).

The region is predicted to bind to empagliflozin in CaMK2G, and PPP1CA. The region has lower activation energy, which indicates that this region is more likely to bind to empagliflozin but does not indicate that binding would alter its catalytic function. Moreover, whether the catalytic process is changed or not requires experimental validation. At this stage, this study can only provide the theoretical basis for the binding of CaMK2G and PPP1CA to empagliflozin, but not experimental validation.

## Discussion

Patients with type 2 diabetes are at increased risk of cardiovascular disease. [1] empagliflozin may reduce mortality from cardiovascular events by affecting hemodynamic effects.

It has been suggested that empagliflozin promotes cardiovascular illness by acting on the CaMKII pathway, decreasing its activity and reducing CaMKII pathway-dependent calcium efflux. However, those genes in which Empagliflozin acts explicitly on the CAMKII pathway have not been reported; therefore, the present study investigated and demonstrated the target genes of Empagliflozin by integrating bioinformatics and molecular docking techniques.

We performed quality control on the 10 experimental sets of data obtained by GEO, and subsequently, we performed differential gene analysis, which yielded 5037 significant differential genes. We then combined with OMIM database NCBI database genecards database to jointly analyze 5 shared genes. We performed functional enrichment analysis and KEGG pathway analysis to determine the biological functions of these five shared genes and the enriched pathways. We concluded that *CaMK2G* and *PPP1CA* are associated with the CaMKII pathway and diabetic cardiomyopathy. The t-test of the validation group showed that these two genes were correlated with the clinical stage of myocardial infarction. Therefore, we concluded that *CaMK2G* and *PPP1CA* could be the target genes of Empagliflozin. Subsequently, we performed a molecular docking study. We verified that the active components of Empagliflozin have minimal binding energy to *CaMK2G* and *PPP1CA*, suggesting that they can bind to each other and that the energy required for binding is small.

Experimental studies in mice showed that activation of *CaMK2G* mediated the inhibition of *ATF4* and *TRB3* and thus improved hepatic insulin signalling [24]. It has also been demonstrated that prostaglandin F 2α promotes hepatic glucose production through the CaMKIIγ/p38/FOXO1 signalling pathway [25]. In mice, adenoviral vector-mediated experiments have shown that the CAMKII pathway regulates vascular smooth muscle cell proliferation and vascular remodelling [26]. Drug Study Shows Reduced Incidence of Cardiovascular Events in Patients with Type 2 Diabetes After Treatment with Empagliflozin [27]. In mice, Empagliflozin significantly reduced the activity of the CaMKII pathway [3]. In the present study, molecular docking analysis showed that the effective molecule of Empagliflozin required very little energy to bind to *CaMK2G*, suggesting that Empagliflozin may indeed interact with the CaMKII pathway-related *CaMK2G* gene and its transcriptional proteins to produce pharmacological effects.

Study shows that calcium is closely associated with vascular smooth muscle cells via the PP1 signalling pathway [28]. *PPP1CA* transcribes protein phosphatase 1 (PP1), a serine/threonine phosphatase. Increased PP1 activity is associated with the development of heart failure [29, 30]. Studies in mice have shown that knocking out the transcribed protein PP1β under the PPP1 gene increases the risk of ventricular remodelling and heart failure [31]. Mouse studies have demonstrated that ATG16L1 promotes autophagy by participating in the phosphorylation of the CSNK2-PPP1 complex, which protects cardiomyocytes. That PPP1 dephosphorylates ATG16L1, leading to autophagy inhibition and induction of apoptosis [32]. These are all studies of the PPP1 pathway associated with cardiovascular events. In the present study, the molecular docking analysis showed that the effective molecule of Empagliflozin required very little energy to bind to *PPP1CA*, suggesting that Empagliflozin may indeed interact with the CaMKII pathway-related *PPP1CA* gene and its transcribed protein to produce its drug effect.

In our study, the CaMKII pathway-associated *CaMK2G PPP1CA* screened by bioinformatics analysis had low activation energy with the active ingredient of Empagliflozin, suggesting that the active ingredient of Empagliflozin is likely to modify the activity of the CaMKII pathway by binding to *CaMK2G PPP1CA* and thus exert its effect on type 2 diabetes and acute cardiovascular events.

## Conclusions

The present study obtained *CaMK2G* and *PPP1CA*, a gene associated with the CaMKII pathway and type 2 diabetes and acute cardiovascular events, by integrative gene analysis. We verified their expression in the relevant dataset, and we concluded that *CaMK2G PPP1CA* could bind to the active ingredient of Empagliflozin at low activation energy by molecular docking technique. We concluded that the clinical effects of Empagliflozin are associated with *CaMK2G* and *PPP1CA* in CaMKII pathway by the above analysis.

## Supporting information

S1 File (RAR)
